# Activity of Selected Bone Formation and Angiogenesis Markers During the Treatment of Limb Length Discrepancy in Children Using Distraction Osteogenesis with the Circular Hexapod External Fixator

**DOI:** 10.3390/jcm14020540

**Published:** 2025-01-16

**Authors:** Oliwer Sygacz, Dominika Miazga, Aleksandra Skorupa, Szymon Stec, Julia Matuszewska, Rafał Kreft, Łukasz Matuszewski, Anna Matuszewska

**Affiliations:** 1 Department of Paediatric Orthopaedics and Rehabilitation, Medical University of Lublin, 20-093 Lublin, Poland; szmn.stec@gmail.com (S.S.); rafalkrzysztofkreft@gmail.com (R.K.); lukasz.matuszewski@umlub.pl (Ł.M.); 2 Students Scientific Association, Department of Paediatric Orthopaedics and Rehabilitation, Medical University of Lublin, 20-093 Lublin, Poland; miazdominika@gmail.com (D.M.); aleksandraskorupa567@gmail.com (A.S.); jmatuszewska39@gmail.com (J.M.); 3 Department of Biochemistry and Biotechnology, Institute of Biological Sciences, Maria Curie-Sklodowska University, 20-614 Lublin, Poland; anna.matuszewska69@gmail.com

**Keywords:** limb lengthening, distraction osteogenesis

## Abstract

**Background/Objectives:** Limb lengthening and deformity correction techniques, particularly distraction osteogenesis, have significantly evolved in pediatric orthopedics. This study examines the temporal changes of key biochemical markers—vascular endothelial growth factor (VEGF), fibroblast growth factor 1 (FGF-1), and the propeptide of type I collagen (P1NP)—during the limb lengthening process. **Methods:** Twenty pediatric patients (aged 13–16) underwent distraction osteogenesis using the Circular Hexapod External Fixator. Peripheral blood samples were collected pre-treatment, three weeks after initiating distraction, and one month post-lengthening. Marker levels were measured using ELISA. **Results:** Serum VEGF concentrations significantly increased during treatment, peaking at T2 (T1 35.91 ± SD 5.54 vs. T2 293.47 ± SD 69.57, *p* < 0.0001), then declined at T3 (293.47 ± SD 69.57 vs. 40.86 ± SD 6.26, *p* < 0.0001). FGF-1 showed minor fluctuations initially but significantly increased by T3 (18.14 ± SD 4.57 vs. 41.56 ± SD 17.15, *p* < 0.01), about 2.3 times higher than baseline. P1NP concentrations exhibited a linear increase, with a significant rise from T2 to T3 (234.06 ± SD 36.57 vs. 280.68 ± SD 35.63, *p* < 0.05), while the T1 to T2 increase was not statistically significant, indicating ongoing osteoblastic activity and bone formation. **Conclusions:** This study highlights the dynamic changes in VEGF, FGF-1, and P1NP during distraction osteogenesis, emphasizing their roles as biomarkers of bone regeneration. These findings enhance the understanding of bone healing mechanisms and could inform future therapeutic strategies for pediatric limb lengthening. Further research is warranted to explore their clinical utility.

## 1. Introduction

Anisomelia, or leg length discrepancy (LLD), is a condition characterized by a significant difference in length between the two lower limbs [1]. Anatomical leg length discrepancies can transpire either congenitally or be acquired over time. Leg shortening may occur directly due to bone loss or indirectly from injuries or infections affecting the epiphyseal growth plate during adolescent development. In cases of congenital or idiopathic leg length discrepancy, the affected limb often grows at a slower rate compared to the unaffected leg [2,3]. Around one-third of the population has a limb length difference ranging from 0.5 cm to 1.5 cm. Even among military recruits, a leg length difference of more than 1.5 cm was identified in 4% of individuals [4]. Leg length discrepancy (LLD) has long been debated as both a biomechanical challenge and a potential contributor to various musculoskeletal issues. LLD has been linked to alterations in gait and running mechanics, standing posture, balance stability, and an elevated risk of conditions such as scoliosis, lower back pain, hip and spine osteoarthritis, aseptic loosening of hip implants, and stress fractures in the lower limbs [5].

The objectives of managing leg length discrepancy (LLD) are to achieve close to normal lower limb lengths and equalize the lengths of both legs after puberty. Various treatment methods have been utilized, including orthotic devices, surgical interventions, and shoe lifts. For discrepancies anticipated to be between 2 and 5 cm, the technique of epiphysiodesis, which involves closing the growth plate, is considered the most effective approach [1,6]. In cases where there is a significant leg length discrepancy or the patient has completed their growth, limb lengthening methods remain applicable.

Limb lengthening and deformity correction techniques used in the pediatric population have evolved significantly over the past several decades. These procedures are commonly performed to correct bone length discrepancies, axial deformities, segmental defects, and impaired or absent bone fusion [7,8,9]. Currently, there is a variety of available devices for limb lengthening and deformity correction, such as conventional or hexapod external fixators, external fixators over nails or rods, and fully implantable intramedullary lengthening nails [10,11,12]. All these methods rely on the distraction osteogenesis phenomenon to reconstruct or lengthen bone tissues [10,13].

Distraction osteogenesis involves several stages. First, an osteotomy is conducted on the bone designated for lengthening. Then, after a short latency period, the ends of the bone are distracted by one of the devices mentioned above. This phase is called the distraction period. It is followed by a long consolidation period to achieve new bone mineralization and remodeling [13]. First described by Alessandro Codivilla at the beginning of the 20th century, the initial results of this method were not promising [14]. Russian orthopedic surgeon Gavriil Ilizarov later completed and improved the technique, significantly elevating results. Ilizarov carefully characterized and optimized the procedure by developing the rigid external ring fixator, precisely determining optimal pin placement and stability within the fixator, determining ideal latency and activation periods, and histologically evaluating the distraction site. These modifications have provided the basis for the highly effective techniques for limb lengthening and deformity correction that are commonly used in pediatric orthopedics today [15,16,17].

The biology of distraction osteogenesis is unique and allows the correction of severe deformities without the need for autogenous bone grafts [7,9,17]. Bone regeneration and mineralization during this process is a sequential cascade, and the two most important parts are osteogenesis and angiogenesis [18,19]. Humoral mechanisms are the main regulators of metabolic processes in the tissues of the limb segment undergoing lengthening. Their active agents are a variety of biomarkers [13,20]. This makes it possible to monitor the regenerative process not only with diagnostic imaging but also at the molecular level by evaluating these factors. Bone turnover markers are important in the evaluation of bone regeneration by providing information on the rate of collagen synthesis and bone healing [13]. Over the past 20 years, they have been widely used in clinical practice and research, with significant progress in their understanding [21].

Among modern markers of bone remodeling, one of the important ones is N-terminal propeptide of type 1 collagen (P1NP), which reflects the synthesis of newly formed type I collagen molecules. P1NP has several advantages, including low diurnal and individual variability and stability at room temperature [22]. It is induced during the synthesis of type I collagen, the major type of collagen in mineralized bone, representing over 90% of the protein in bone [23]. Osteoblasts synthesize collagen in the form of procollagen—a precursor that contains a short signaling sequence and terminal elongation propeptides that comprise the P1NP and the propeptide on the carboxyl end. These terminal propeptides are cleaved by specific proteinases before the formation of final collagen molecules. Both molecules enter circulation, and their concentrations provide a reliable measure of type I collagen synthesis activity. Although other tissues, such as skin and tendons, also contain type I collagen, their turnover rates are slower than that of bone and their contribution to circulating P1NP levels is minimal. This specificity makes P1NP a highly useful marker for monitoring bone turnover in clinical practice [21,22].

Equally important are the growth factors involved in bone healing and regeneration, such as vascular endothelial growth factor (VEGF) and fibroblast growth factor (FGF). These factors play a critical role in promoting angiogenesis, cell proliferation, and tissue repair, all of which are essential for effective bone regeneration [24,25].

The vascular endothelial growth factor (VEGF) is one of the most powerful inducers of angiogenesis and plays an important role in regulating collagen fiber formation [26,27]. VEGF, identified as comprising the most significant molecules involved in vascular development, is necessary for proper bone development [28]. An intact local microcirculation is essential for effective bone repair. Several studies suggest an interdependence between vascular and skeletal tissues, each providing morphogenetic signals and environmental cues essential for the development of the other. Communication between blood vessels and bone cells must be tightly coordinated to ensure their close proximity during physiological changes in bone turnover and repair processes [29].

Fibroblast growth factor (FGF) plays an important role in bone remodeling regulation. FGF acts as an active mitogen stimulating cell proliferation, promoting collagen synthesis, and providing protective functions under conditions of cellular stress. A unique feature of these molecules is their interaction with heparin and proteoglycans, including heparan sulfate, which stabilizes these proteins [30]. In addition, they are involved in regulating the production of alkaline phosphatase (ALP), an enzyme critical for bone mineralization. Notably, FGF suppresses the expression of the ALPL gene, resulting in decreased bone ALP activity. This leads to increased pyrophosphate levels and decreased phosphate availability, affecting bone mineralization processes [21].

Bone formation and resorption processes are closely linked to one another and remain in balance among healthy individuals. The measurement of biochemical markers of bone remodeling provides a global picture of the simultaneous processes occurring throughout the skeleton [31].

Bone turnover markers are important in the evaluation of bone regeneration by providing information on the rate of collagen synthesis and bone healing.

These findings suggest that distraction osteogenesis is in part supported by both angiogenesis and an increased activity of bone formation markers [32,33]. By achieving a more comprehensive understanding of the dynamics of change in the aforementioned markers during distraction osteogenesis, it would be possible to more effectively regulate the treatment process, thereby enhancing the safety and efficacy of limb-lengthening procedures in pediatric patients. The study aims to investigate the molecular mechanisms of bone regeneration during distraction osteogenesis by examining changes in P1NP, VEGF, and FGF, focusing on the interaction between osteogenesis and angiogenesis. Evaluating biochemical markers could establish their use as an early and reliable indicator of bone healing, complementing traditional imaging methods. By identifying key trends and correlations, the research will optimize treatment protocols, improve clinical outcomes, and ensure more effective bone healing in pediatric patients.

## 2. Materials and Methods

### 2.1. Patients

A cohort of 20 unrelated young patients aged 13–16 participated in the study. All patients were Caucasian and of Polish descent, originating from the eastern region of Poland. All of them were in good health with no concurrent diseases. The study group included twelve girls and eight boys, among whom the lengths of sixteen tibiae and four femurs were decreased. They were admitted to the Orthopedics and Rehabilitation Department for the diagnosis and treatment of limb length discrepancy. Each patient had a cross-sectional X-ray of the lower limbs. The difference in limb length was 3.2–5.7 cm (on average 4.53 ± SD 0.72). The principles underlying distraction osteogenesis, as initially established by Ilizarov, were followed throughout the study. The multiple drill hole technique for osteotomy in the distal metaphyseal region of the femur or the proximal metaphyseal region of the tibia was performed according to the deformity. A Circular Hexapod External Fixator was applied to the patients. After bone alignment, the positioning of the screws, and the initial setting of the external device were assessed and confirmed. Patients underwent X-ray imaging. The average daily distraction rate was maintained at 0.7 mm for the tibia and 1 mm for the femur, with continuous monitoring of measurements over a 24 h period. The lengthening lasted an average of 9.24 ± SD 1.21 weeks. During the lengthening phase, partially loaded gait (25%) with the aid of crutches was recommended as tolerated. All patients were followed up every two weeks until the end of the lengthening phase. Additionally, patients were followed up in the third week after the initiation of the procedure, and a blood sample was taken. Radiological confirmation of properly consolidated bone callus formation was defined as three cortices visible in two perpendicular projections. After consolidation was confirmed by X-ray, the external fixator was removed, and the patient was able to fully load the limb. In the group, we had one complication: delayed consolidation of the femur.

Written informed consent for genetic studies was secured from all the patients and subjects from the study group. The study protocol was evaluated and approved by the Ethics Committee at the Medical University in Lublin (KE-0254/134/2018).

### 2.2. Biochemical Marker Concentration

Peripheral blood samples were collected from all participants at three distinct time points during the study. The initial sample was collected one week before the limb lengthening commencement procedure (T1). The second sample was taken three weeks after the procedure began (T2), while the final sample was collected one month post-completion of the limb lengthening intervention (T3).

ELISA immunoenzymatic assays were used to determine the concentration of selected factors: VEGF, FGF-1, and P1NP. All necessary assays were purchased from Cloud-Clone Corp. (Address: 23603 W. Fernhurst Dr., Unit 2201, Katy, TX 77494, USA). The Human VEGF solid-phase sandwich ELISA (ELISA Kit for Vascular Endothelial Growth Factor A (VEGF), Product No.: SEA143Hu) was used to determine the concentration of VEGF. A target-specific antibody is pre-coated in the wells of the supplied microplate. Samples, standards, and controls are then added into these wells and bind to the immobilized (capture) antibody. The sandwich is formed by the addition of the second (detector) antibody, and a substrate solution is added that reacts with the enzyme-antibody-target complex to produce a measurable signal. The intensity of this signal is directly proportional to the concentration of the target present in the original specimen. The Procollagen Type 1 N-Terminal Propeptide (P1NP) ELISA Assay Kit (Enzyme-linked Immunosorbent Assay Kit For Procollagen I N-terminal Propeptide (P1NP), Product No.: CEA957Hu) was used to assess concentration of P1NP. The immunoplate is pre-coated with a monoclonal antibody specific for human P1NP. Standards and samples are pipetted into the wells and any human P1NP present is bound by the immobilized antibody. After washing away any unbound substances, a biotin-labeled polyclonal antibody specific for human P1NP is added to the wells. After the wash step to remove any unbound reagents, streptavidin-HRP conjugate (STP-HRP) is added. After the last wash step, an HRP substrate solution is added and the color develops in proportion to the amount of human P1NP bound initially. The assay is stopped, and the optical density of the wells is determined using a microplate reader. ELISA Assay Kit for Fibroblast Growth Factor 1 (FGF-1, Product No.: CEA957Hu) was used to assess concentration for human FGF-1 level. The FGF-1 ELISA was performed according to the manufacturer’s protocol. Samples were added to a 96-well plate pre-coated with an FGF-1 capture antibody, followed by incubation with a biotinylated detection antibody and HRP-conjugated streptavidin. After substrate addition, the colorimetric reaction is stopped, and absorbance is measured at 450 nm using a microplate reader to quantify FGF-1 concentrations.

### 2.3. Statistical Analysis

Statistical analysis of the results was carried out. To present the results on a quantitative scale, methods of descriptive statistics were used, i.e., arithmetic mean, standard deviation (SD). In order to assess the conformity of the distribution of the studied variables to a normal distribution, the Shapiro–Wilk test was applied. A one-factor analysis of variance test was used with repeated measurements (with Tukey’s post-hoc test) or Friedman’s analysis (with Dunn’s post-hoc test). For VEGF and P1NP concentrations, the Shapiro–Wilk test indicated a normal distribution, so Tukey’s post-hoc test was used. In contrast, for FGF-1, the Shapiro–Wilk test indicated a non-normal distribution, so Friedman’s analysis was applied, followed by Dunn’s post-hoc test. Results were considered statistically significant at *p* < 0.05. The analysis was performed using GraphPad Prism v.9.0 software.

## 3. Results

Baseline values represent the mean of the twelve measurements taken at each blood sample. Averaged results of the measurements are presented in Table 1 and the figures.

### 3.1. VEGF

**Figure 1:**
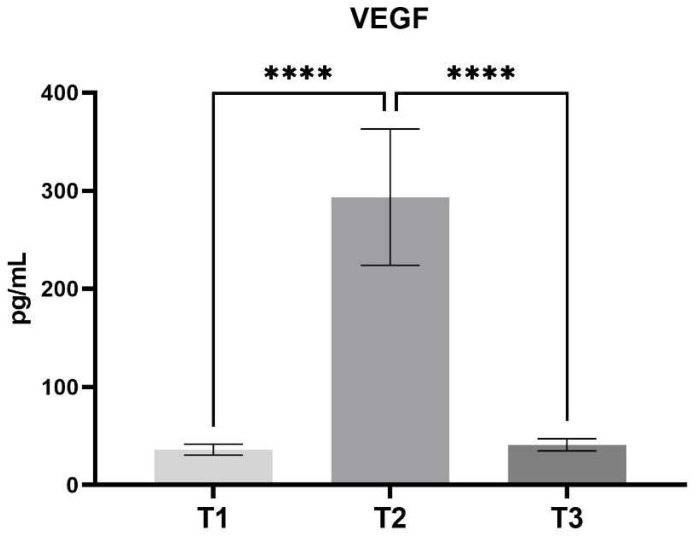
Concentration of VEGF between different stages of the study. (Mean, SD) T1—pre-operative measurement, T2—measurement during lengthening, T3—measurement after lengthening. ****—*p* < 0.0001.

The time course of serum VEGF concentrations exhibited an increase following the treatment, with concentrations maintained at a level approximately six-fold higher than that observed prior to treatment (T1—35.91 ± SD 5.54 vs. T2—293.47 ± SD 69.57, *p* < 0.0001). Throughout the course of the treatment procedure, serum VEGF concentrations demonstrated a gradual decline (T2—293.47 ± SD 69.57 vs. T3—40.86 ± SD 6.26, *p* < 0.0001). However, they remained elevated but not statistically significant until the conclusion of the study period. One month after the conclusion of treatment, samples exhibited slightly elevated but not statistically significant VEGF concentrations (T1—35.91 ± SD 5.54 vs. T3—40.86 ± SD 6.26) in comparison to the initial values (Figure 1).

### 3.2. FGF-1

**Figure 2:**
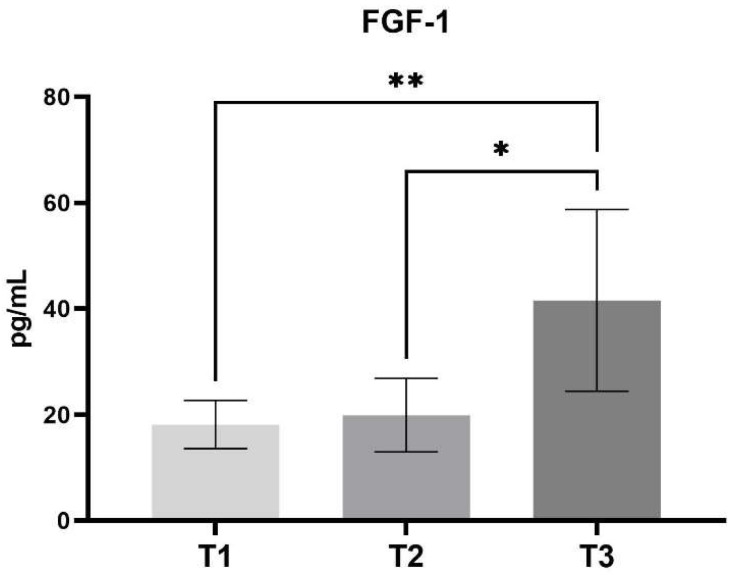
Concentration of FGF-1 between different stages of the study. (Mean, SD) T1—pre-operative measurement, T2—measurement during lengthening, T3—measurement after lengthening. *—*p* < 0.05, **—*p* < 0.01.

In contrast to the notable alterations in VEGF levels post-surgery, FGF-1 serum concentrations demonstrated minor fluctuations. During the treatment phase, there was an incremental but not statistically significant increase in FGF-1 concentration in comparison to the preoperative state (T1—18.14 ± SD 4.57 vs. T2—19.9 ± SD 6.94). However, at the conclusion of the treatment period, the concentration reached a considerable peak, approximately 2.3 times the baseline and source value (T1—18.14 ± SD 4.57 vs. T3—41.56 ± SD 17.15, *p* < 0.01) (Figure 2).

### 3.3. P1NP

**Figure 3:**
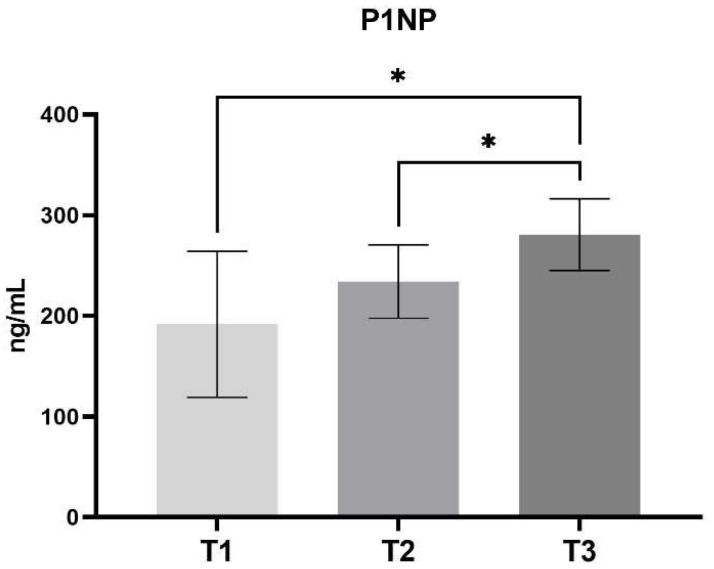
Concentration of P1NP between different stages of the study. (Mean, SD) T1—pre-operative measurement, T2—measurement during lengthening, T3—measurement after lengthening. *—*p* < 0.05.

In light of the aforementioned factors, the concentration of P1NP demonstrated the least degree of fluctuation throughout the course of this study. It is noteworthy that the concentration of this factor increased in a linear fashion. A comparison of the successive stages reveals an increase in the P1NP value of 42–46 ng/mL with each stage (T1—191.66 ± SD 72.51 vs. T2—234.06 ± SD 36.57 with no statistical difference and T2—234.06 ± SD 36.57 vs. T3—280.68 ± SD 35.63, *p* < 0.05) (Figure 3).

In conclusion, the results demonstrated a statistically significant increase in vascular endothelial growth factor (VEGF) levels during treatment, reaching a value of 293.47 ± SD 69.57 pg/mL. Conversely, fibroblast growth factor (FGF-1) and type I N-terminal procollagen propeptide (P1NP) levels reached their highest values following treatment, at 41.56 ± SD 17.15 pg/mL 280.68 ± SD 35.63 ng/mL.

## 4. Discussion

Recent studies have revealed that the molecular signaling pathways are crucial in linking induced strain to bone regeneration. The molecular signals involved in the regenerative process during distraction osteogenesis (DO) closely resemble those seen in fracture healing. These signals include pro-inflammatory cytokines, members of the transforming growth factor-beta (TGF-β) superfamily, and angiogenic factors [34,35]. Humoral mechanisms regulate metabolic processes in lengthening limb tissues via numerous growth factors and bone markers [20]. Levels of biomarkers depend on the stage of bone formation and the lengthening itself. In distraction osteogenesis, mechanical forces have been shown to increase the levels of specific osteoinductive factors during bone healing. The increase in these regulatory molecules in response to distraction forces may be the result of either stimulation of local production or activation of pre-existing factors from their latent forms in the extracellular bone matrix, reflecting local bone healing mechanisms in the blood circulation [33]. Research suggests that metabolic processes associated with surgical lengthening may also be influenced by the etiology of limb length discrepancies, potentially affecting the new bone formation [20]. While numerous studies have measured the levels of growth factors in experimental models of distraction osteogenesis, there is still a wide gap in research focused on the human population. This deficiency of information emphasizes the importance of conducting further research on this matter [20]. The results of this study provide important insights into the dynamic responses of key biomarkers, such as vascular endothelial growth factor (VEGF), fibroblast growth factor 1 (FGF-1), and procollagen I N-propeptide (P1NP), before, during, and after treatment. These findings have a significant impact on understanding the processes involved in distraction osteogenesis.

### 4.1. VEGF

Angiogenesis is widely recognized as one of the critical processes involved in the formation of new bones [19]. It is regulated by interacting with molecules that control neovascularization [33,36]. One of the most important factors here is the vascular endothelial growth factor (VEGF), which plays a key role in promoting the formation of new blood vessels and is regulated by multiple elements, including growth factors, transcription factors, hormones, and mechanical stimulation. Additionally, VEGF promotes the transport of osteogenic cells, nutrients, oxygen, and minerals necessary for bone mineralization to the site of new bone formation [19,37]. In this study, we observed a six times higher serum level of VEGF during the distraction phase. This notable increase is coherent with the known role of VEGF. Although VEGF levels dropped over time, they remained significantly elevated at the end of the study compared to baseline. Notably, serum VEGF levels were still slightly elevated (approximately 5 pg/mL above baseline) even one month after treatment. Studies suggest that the elevated levels of VEGF may be related to the body’s stress response. For example, the initial peak in VEGF levels may be related to the trauma caused by the surgical procedure, as has been observed in studies that examined VEGF levels during the post-traumatic healing of bone fractures among surgically treated patients [33]. More interestingly, a greater degree of vascularization is observed in the regenerating tissues of a distraction osteogenesis model compared to fracture healing, even though both fracture repair and bone formation during distraction osteogenesis require increased blood flow [32]. Moreover, studies suggest that the higher increase in VEGF observed in distraction osteogenesis is due to other mechanical stresses which stimulate VEGF secretion, rather than trauma or postoperative inflammatory responses [33]. As VEGF is a key stimulator of angiogenesis, it is assumed that exogenous VEGF administration could stimulate angiogenesis and improve bone formation. VEGF was injected locally into the distraction gap during the distraction phase in a murine tibia nonunion model. Measurements taken 27 days after the end of distraction showed a greater volume of new bone. VEGF also regulates osteoclast differentiation and migration; however, overly high levels of VEGF can lead to an over-recruitment of osteoclasts, potentially leading to excessive resorption of newly formed bone [19].

### 4.2. FGF-1

The mechanical forces applied during distraction osteogenesis activate a biological response involving several growth factors that enhance and accelerate bone formation [38]. These include fibroblast growth factors, of which FGF-1, FGF-2, and FGF-18 are especially important for bone development and repair [39]. It is likely that their expression is a direct consequence of the distraction process. However, their expression during the first week of consolidation suggests that these factors may continue to be produced as a result of the mechanical forces exerted during the distraction phase. In addition, it is possible that other unidentified cytokines may play a role in the consolidation phase [40]. FGFs belong to a family of heparin-binding growth factors [24]. They can bind to heparin or heparan sulfate proteoglycans, which are low-affinity receptors that regulate the binding and activation of FGFRs. Notably, in vivo studies have shown that FGF-1 alone, when delivered via composite implants, can induce de novo bone formation [30]. Inactivity of FGFR1 has been proven to increase bone mass, suggesting that signaling through this receptor negatively regulates osteoblast maturation and bone formation in vivo [31]. The FGF signaling pathway has been studied for its role in regulating the proliferation and differentiation of osteoblasts and fibroblasts, contributing to osteogenesis and several other critical cellular processes, including angiogenesis and wound healing. It plays a crucial role in both intramembranous and endochondral ossification in osteoprogenitor cells [24]. In this study, FGF-1 showed moderate changes in concentration during treatment, with a progressive increase culminating in a significant peak at the end of the study, reaching 2.3 times the baseline value. The delayed peak in FGF-1 levels observed may reflect its role in the later stages of tissue regeneration, particularly in extracellular matrix remodeling and epithelialization. The existing literature on FGF-1 dynamics during distraction osteogenesis is limited. One study reported the highest expression of FGF-1 in 50–75% of chondrocytes during the fifth week after the initiation of lengthening, coinciding with the first week of the consolidation phase when limb lengthening ceased. However, this finding is not directly comparable to the present study, as it was based on immunohistochemical analysis of cellular FGF-1 expression rather than serum concentrations [40]. Additionally, studies evaluating FGF-1 levels during fracture treatment provide interesting insights. One study found that FGF-1 did not peak until two weeks post-fracture compared to other factors, but follow-up levels were not measured, leaving uncertainty about whether concentrations continued to rise or began to decrease [41]. These gaps highlight the need for further investigation into the temporal dynamics of FGF-1 during both fracture repair and distraction osteogenesis to fully understand its role in bone healing. In our study, FGF-1 levels were measured three weeks after the start of lengthening. It is possible the peak occurred in subsequent weeks, as reported by Haque et al., who observed the highest expression of FGF-1 in cells one week after the cessation of lengthening, during the consolidation phase [40]. FGF-1 is known to play multiple roles in biological processes, including angiogenesis, wound healing, bone healing, and neuroprotection [30]. It is produced by fibroblasts, which are important contributors to the later stages of repair and bone remodeling during fracture healing. Fibroblast-produced proteins, such as collagen, elastin, reticular fibers, and organic matrix, are critical for fracture healing, underscoring the potential importance of FGF-1 in bone regeneration and remodeling [42,43].

The increase in FGF-1 levels at the end of the lengthening phase may be influenced by several factors, including wound healing associated with the removal of the external device and the cumulative effects of treatment-related changes in the limb. The significant increase in FGF-1 at the end of treatment suggests that this growth factor plays a critical role in post-treatment tissue maintenance and repair, complementing the earlier angiogenic effects driven by VEGF. In conclusion, these findings highlight the importance of further research to better understand the mechanisms that regulate FGF-1 dynamics during and after distraction osteogenesis. Enhanced knowledge in this area is essential to optimize treatment strategies and improve clinical outcomes.

### 4.3. P1NP

P1NP serves as a specific marker of bone formation and osteoblast activity, and its concentration is proportional to the amount of newly synthesized collagen in bone tissue. Children and adolescents typically have significantly elevated bone marker levels due to fast skeletal growth and rapid bone turnover during puberty development [44]. P1NP plays a critical role in monitoring bone turnover in conditions where bone growth may be dysregulated, such as bone malignancies and prolonged immobilization [45]. It has also been widely utilized to assess bone turnover in several conditions, including pediatric osteoporosis, osteogenesis imperfecta, and growth hormone deficiency [46]. P1NPs follow specific trends during the normal fracture healing process. Abnormalities in P1NP levels may indicate delayed healing or nonunion. Studies of tibial and femoral shaft fractures have shown that P1NP levels peak between four and twelve weeks after fracture, with elevated levels persisting for up to one year. Among patients with delayed union after tibial fractures, P1NP levels were lower at eight, twelve, and twenty-four weeks. This suggests that P1NP may allow earlier detection of delayed fracture healing than radiographs taken at the three-month mark [47]. Of the markers measured, P1NP showed the lowest fluctuation in this study. However, its consistent linear progression, with an increase of 42–46 ng/mL at each stage, suggests a continuous process of collagen formation and bone turnover throughout treatment. This observation is supported by previous research identifying P1NP as a reliable marker of bone formation, particularly in studies related to fracture healing and bone regeneration.

### 4.4. Limitations

The present study has several limitations. Firstly, the study cohort was relatively small, comprising only 20 participants, and the absence of a control group raises concerns about the generalizability and accuracy of the results. Secondly, the scope of the study could have been broader, as distraction osteogenesis is influenced by various factors that were not considered in this investigation. Nevertheless, future investigations may require a more comprehensive approach in this regard

## 5. Conclusions

This study highlights the dynamic roles of VEGF, FGF-1, and P1NP as key biomarkers in distraction osteogenesis, reflecting different phases of bone formation. VEGF plays a critical role in promoting angiogenesis and bone formation, peaking during the distraction phase. FGF-1 shows a delayed increase, indicating its involvement in the later stages of tissue regeneration and extracellular matrix remodeling, while P1NP exhibits a steady increase, reflecting ongoing collagen synthesis and bone turnover. Together, these biomarkers serve as potential molecular indicators for monitoring bone healing, complementing imaging techniques and advancing the understanding of the interplay between angiogenesis and osteogenesis. Future research should include larger, more diverse cohorts, including patients with nonunion, malunion, or delayed union, to identify differences in biomarker trends and molecular signatures associated with complications. Research on the impact of comorbidities such as diabetes and osteoporosis should also be considered, as this may facilitate the development of personalized treatment approaches. Further investigation of the therapeutic potential of exogenous VEGF, FGF-1, or synthetic analogs may enhance angiogenesis and bone regeneration, particularly in cases of impaired healing. Extending biomarker monitoring into the late consolidation phase may provide insights into long-term bone remodeling and stabilization. Integrating biomarker analysis with advanced imaging techniques could improve diagnostic accuracy by correlating molecular and structural healing processes. Further investigations are warranted to ascertain their clinical relevance and potential therapeutic applications.

## Figures and Tables

**Table 1:** Concentration of VEGF, FGF-1, and P1NP between different stages of the study. T1—pre-operative measurement, T2—measurement during lengthening, T3—measurement after lengthening.

Stage of the Study	T1	T2	T3
VEGF [pg/mL]	35.91 ± SD 5.54	293.47 ± SD 69.57	40.86 ± SD 6.26
FGF-1 [pg/mL]	18.14 ± SD 4.57	19.9 ± SD 6.94	41.56 ± SD 17.15
P1NP [ng/mL]	191.66 ± SD 72.51	234.06 ± SD 36.57	280.68 ± SD 35.63

## Data Availability

Data are available in the Department of Paediatric Orthopaedics and Rehabilitation, Medical University of Lublin, Lublin, Poland (medical documentation).
